# Segmenting Mechanomyography Measures of Muscle Activity Phases Using Inertial Data

**DOI:** 10.1038/s41598-019-41860-4

**Published:** 2019-04-03

**Authors:** Richard B. Woodward, Maria J. Stokes, Sandra J. Shefelbine, Ravi Vaidyanathan

**Affiliations:** 1 Imperial College London, Department of Mechanical Engineering, London, UK; 2 University of Southampton, Faculty of Health Science, Southampton, UK; 3 Northeastern University, Department of Mechanical and Industrial Engineering, Boston, MA USA; 4 Northeastern University, Department of Bioengineering, Boston, MA USA

## Abstract

Electromyography (EMG) is the standard technology for monitoring muscle activity in laboratory environments, either using surface electrodes or fine wire electrodes inserted into the muscle. Due to limitations such as cost, complexity, and technical factors, including skin impedance with surface EMG and the invasive nature of fine wire electrodes, EMG is impractical for use outside of a laboratory environment. Mechanomyography (MMG) is an alternative to EMG, which shows promise in pervasive applications. The present study used an exerting squat-based task to induce muscle fatigue. MMG and EMG amplitude and frequency were compared before, during, and after the squatting task. Combining MMG with inertial measurement unit (IMU) data enabled segmentation of muscle activity at specific points: entering, holding, and exiting the squat. Results show MMG measures of muscle activity were similar to EMG in timing, duration, and magnitude during the fatigue task. The size, cost, unobtrusive nature, and usability of the MMG/IMU technology used, paired with the similar results compared to EMG, suggest that such a system could be suitable in uncontrolled natural environments such as within the home.

## Introduction

Muscle monitoring is of great interest in areas such as clinical assessment, rehabilitation, and sport science. Understanding, ideally quantifying, when a muscle state (e.g., relaxed, active, or fatigued) has changed in relation to whole body motion can assist in physical training and muscle conditioning. Furthermore, recognising the onset of muscle fatigue can act as an indicator to limit exertion, thus preventing injury or other negative health impact.

Monitoring of muscle activity is predominantly achieved through electromyography (EMG), a method which records the electrical response from contracting muscle fibres. While still the ‘gold standard’ EMG also has well documented disadvantages including change in signal response because of skin impedance changes (e.g., during perspiration), need for electrical connection to the skin, level of hardware required for collection (amplifiers, etc.), are typically expensive, non-portable, and require training and knowledge for sensor placement and operation, which reduces its applicability in use outside of a laboratory^1–3^. These disadvantages, which can be managed in a controlled environment, can be difficult to handle in pervasive settings (monitoring in a natural environment). Despite these disadvantages, EMG is still widely used, even if EMG data are sometimes sub-optimal. Some of the disadvantages can be alleviated by using dry electrodes or other EMG collection methods, which are popular in portable applications like myoelectric control^4^. An alternative muscle monitoring technique, mechanomyography (MMG), differs from EMG in that it measures the low-frequency (2–200 Hz) mechanical response of the lateral oscillation of muscle fiber during contraction^5^. MMG offers some potential benefits over EMG, including exemption from skin impedance changes, a higher signal-to-noise ratio, and a lower sensitivity to sensor placement on the muscle of interest^6–8^, however lack of established sensors and acoustic/vibrational interference have inhibited its mainstream use. Muscle monitoring is well documented for both EMG and MMG, each producing complementary results^9–11^, suggesting that MMG could be used as an alternative technology in muscle monitoring.

An understanding of how muscle activity relates to whole body motion is critical in many monitoring applications. However, identifying activities or repetitions/training sets from muscle activity data is also a non-trivial task. Segmenting data into specific tasks can be achieved by using additional information from strain gauges, dynamometers, goniometers, cameras, or estimated with *a priori* knowledge^12^. These techniques further limit the use of monitoring outside of a controlled environment, as they either restrict range of motion or practical use, or are large, immobile, and expensive. An alternative to these laboratory-based techniques is therefore needed so that motion and muscle activity can be simultaneously measured in a pervasive (natural) environment.

The present paper demonstrates use of an inexpensive, unobtrusive, sensor package, capable of monitoring muscle activity and change during a physical task. This is achieved using a custom MMG sensor and an inertial measurement unit (IMU), packaged into a strap worn around the thigh. To the best of our understanding, the unique combination of MMG and IMU technologies, as well as their application shown here, is novel and has not yet been demonstrated. The objective of this study was to segment muscle activity phases and compare MMG and EMG measurements of muscle activity during an exerting task, to demonstrate measures of muscle activity that could be performed in a natural environment.

## Results

### Pre- and Post-Exertion

**Figure 1:**
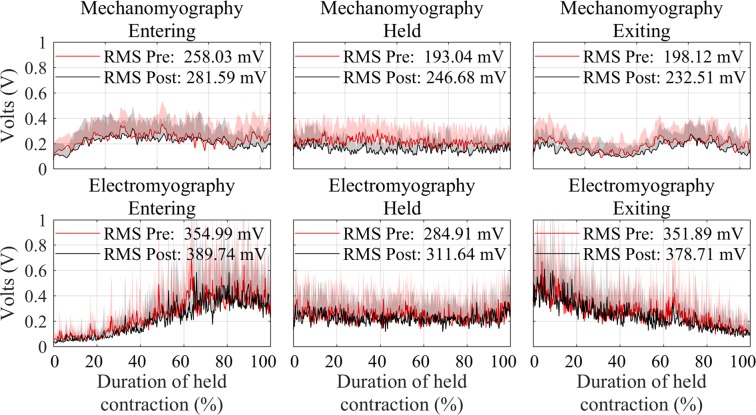
Grouped participant data showing comparison of pre (red) and post-exertion (black) RMS results for MMG (top row) and EMG (bottom row). Data have been divided into entering the squat (left column), holding the squat (middle column), and exiting the squat (right column). EMG is hardware amplified whereas MMG has no amplification. Standard deviation is plotted as a shaded envelope.

Comparisons between pre- and post-exerting activity saw an increase in root mean square (RMS) amplitude in all contraction periods (entering, held, and exiting the squat) for both MMG and EMG, with an average percentage increase of 18.09% and 9.79%, respectively (Table 1). No significant difference (*P* > 0.05) was found post-exertion for entering, held, nor exiting for both EMG and MMG RMS results. Figure 1 shows a comparative result of each contraction period for the RMS results, for both MMG and EMG, pre- and post-exertion.

**Table 1:** Results of before (pre) and after (post) exertion task. Raw values (V/Hz) and percentage change (%) of RMS and MPF between pre and post exertion, in each of the contraction periods for both MMG and EMG, are shown.

			Entering		Held		Exiting		Average	
			Pre	Post	Pre	Post	Pre	Post	Pre	Post
MMG	RMS	V	0.26	0.28	0.19	0.25	0.20	0.23	0.22	0.25
		%	7.69		31.58		15.00		18.09	
	MPF	Hz	7.42	8.87	8.12	8.98	7.51	7.74	7.68	8.53
		%	19.54		10.59		3.06		11.06	
EMG	RMS	V	0.38	0.43	0.46	0.50	0.40	0.43	0.41	0.45
		%	13.16		8.70		7.50		9.79	
	MPF	Hz	82.39	84.91	90.07	93.51	92.65	94.61	88.37	91.01
		%	3.06		3.82		2.12		3.00	

All results showed no significant difference (*P* > 0.05) post exertion.

**Figure 2:**
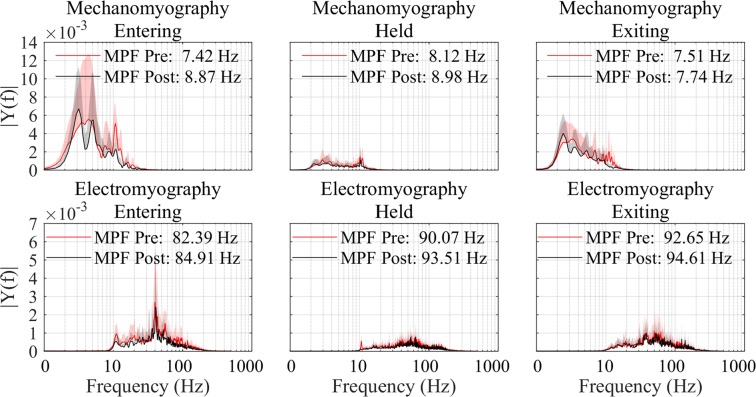
Grouped participant data showing comparison of pre (red) and post-exertion (black) MPF results for MMG (top row) and EMG (bottom row). Data have been divided into entering the squat (left column), holding the squat (middle column), and exiting the squat (right column). Standard deviation is plotted as a shaded envelope.

MMG mean power frequency (MPF) saw an increase of 11.06% 10 minutes post-activity, whereas EMG MPF saw a 3.00% increase (Table 1). No significant difference (*P* > 0.05) was found post-exertion for entering, held, nor exiting for both EMG and MMG MPF results. Figure 2 shows an equivalent comparative result for the MPF results, pre- and post-exertion.

Average knee flexion angles across all participants were estimated at 64.11° and 64.25° for pre- and post-exertion respectively. The Wilcoxon signed rank test reported an achieved confidence of 94.09% for the data analysed.

### During Exertion

**Figure 3:**
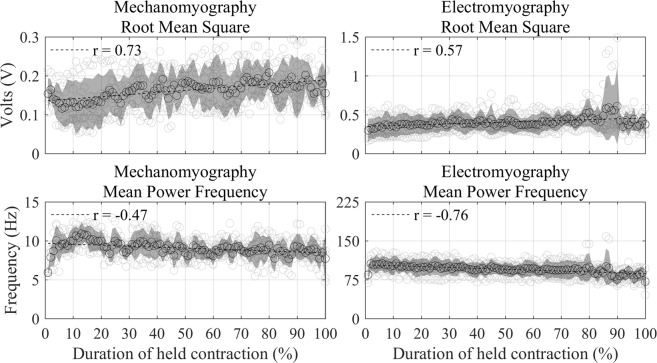
Grouped participant data showing significant (*P* < 0.01) changes in RMS (top row) and MPF (bottom row) for both MMG (left column) and EMG (right column) during the sustained exerting task. Standard deviation is plotted as a shaded envelope, as well as linear lines of best fit and the calculated Pearson’s correlation coefficients.

On average, the participants lasted 88.5 seconds (28.48 standard deviation) while maintaining the wall squat. MMG RMS and EMG RMS show a significant (*P* < 0.01) positive linear relationship against time during the wall squat activity, with an RMS increase of 29.35% and 15.20% for MMG and EMG respectively. MMG MPF decreased 5.98% in frequency during the activity and resulted in a moderate linear negative correlation against time, albeit still significant (*P* < 0.01). EMG MPF declined significantly (*P* < 0.01) throughout the wall squat, with a strong negative linear relationship and an 18.07% decrease on average. Figure 3 shows the change in RMS and MPF for both MMG and EMG during the sustained exerting activity, represented as a percentage of average duration.

Average knee flexion across all participants were estimated at 78.44° during the sustained exerting activity.

## Discussion

The present study has shown how combining information from muscle contraction (MMG and EMG) and dynamic movement (IMU) can enable a better understanding of human muscle and motion activity at specific periods of contraction. This study was designed to highlight how an IMU can be used in segmentation of contraction periods for a more refined insight into how muscle amplitude and frequency change at specific points of an activity; demonstrated with a squatting task seen here. Furthermore, this study tracked changes in mechanical (MMG) and electrical (EMG) muscle activity of rectus femoris during different phases of a fatiguing activity task using the squat in order to demonstrate this technology for potential pervasive use. Using an IMU, squatting repetitions were identified and segmented into the three stages of contractile activity; entering, held, and exiting the squat. These phases of activity involved combinations of static, dynamic, concentric, and eccentric muscle activity.

An elevated RMS and MPF were seen post-exertion for both EMG and MMG, when compared against pre-exertion measures, however, these were not found to be significantly different. Greater changes in signal magnitude may suggest MMG has a better response to changes in muscle force when compared to EMG, whereas the change in frequency may suggest a higher sensitivity to changes in motor unit recruitment, although the lack of statistical significance cannot fully verify this claim. Alternatively, the change could be influenced by any skin or motion artefacts or sensor drift. Although these were controlled to the best of our ability it is plausible that the results were attributed by these disturbances.

RMS values seen in Fig. 1 show that MMG had an overall lower signal amplitude when compared to EMG, although it is important to note that MMG data were not amplified, whereas EMG data were, further confirming that MMG has a larger SNR than EMG as reported in literature^13^.

MPF and frequency information presented in Fig. 2 show peak frequencies around 7–8 Hz for MMG and 80–90 Hz for EMG respectively, which falls within the expected ranges for the quadriceps^14,15^. Most literature suggests a lower or equal MPF result for both EMG and MMG data post exertion/fatigue^9–11^. Results seen here show the opposite, although the same literature stresses that this relationship is complex and relies on a variety of conditions such as muscle type, level of fatigue, MVC level, length of monitoring time and time allowed post-fatigue, and inter-participant/gender differences^9–11^. The differences seen in the present study are expected to be a result of these variations, and the higher MPF is believed to be due to a larger number of muscle fiber recruitment following the fatiguing task.

Changes during the exerting activity of a sustained wall squat showed a significant increase in RMS and significant decrease in MPF over time for both EMG and MMG. While MMG showed a greater change and stronger Pearson’s correlation coefficient over time against EMG, the opposite is true for the frequency results, showing EMG had a greater decline in MPF against MMG. Again, this is expected from the literature, as EMG reflects the electrical activity of muscle, which needs to adapt during sustained contraction to compensate for the loss of Type II motor neuron activity and progressive change to predominantly Type I motor neurons, which have lower firing frequencies^16^. Conversely, MMG reflects the mechanical output of muscle and may therefore be less sensitive to detecting the frequency-specific components of muscle activity than EMG. However, MMG has shown an ability to detect differences between fast (Type II) and slow (Type I) muscle fiber types^17^. Much like the pre/post exertion results, MMG had a greater response to amplitude change than EMG, despite MMGs lack of amplification.

A few studies have examined at the level of voluntary contraction verses knee flexion in the quadriceps and hamstring muscles during squats. The quadriceps have been found to contract at 49% MVC between 60°–90° (entering squat), and 32% MVC between 90°–60° (exiting squat)^18^. Another study has reported similar results with a 40% MVC at 60° knee flexion for quadriceps during a held isometric squat^19^. Results seen here show that the participants’ squat angles were between 64 and 78°, suggesting that the present study’s activities involved a low MVC task.

Many studies have demonstrated an inverse relationship between EMG amplitude and frequency during sustained isometric fatiguing activity, regardless of maximum voluntary contraction (MVC) intensity^10,11,20^. MMG, however, has a more complex relationship, with results suggesting an increase over time in MMG amplitude in low intensity contractions (~5–50% MVC)^10,20–25^, stable in medium intensity contractions (~50–75% MVC)^7,10,25,26^ and a decrease over time in high intensity contractions (~75–100% MVC)^10,11,24,25,27^, during sustained isometric fatiguing activity. Equivalent responses from MMG frequency components are less complex, with literature stating the MPF declines or fluctuates over all intensity contractions over time during sustained isometric fatiguing activities^10,11,20,21,23–25,27–29^. Although variations are found in amplitude and frequency components in fatiguing studies, literature continues to agree on a strong correlation of MMG amplitude against voluntary force^5,7,30^. The present study recognised a significant decline in both MMG and EMG MPF during the sustained contraction. The decline in frequency is suggested to be due to reducing motor unit firing rate in response to the low MVC contraction force estimated in the present study^10^, as well as a decline in the number of muscle fibres recruited, which is known to occur with EMG as the muscle becomes fatigued^9^.

The majority of MMG studies to date have focused on isometric contractions but dynamic contractions have also been studied, reviewed by Ibitoye *et al*.^31^. Studies suggest that the MMG signal follows force changes more closely than the EMG signal, as it reflects mechanical activity^5,9,11,32,33^. However, MMG signal amplitude and frequency responses differ throughout the literature, and these components depend largely on the muscle being examined and the fatiguing protocol used^34^.

Unlike more static systems, the IMU/MMG device relies on battery power and therefore can only be used for a limited time. The battery life for this device is around 10 hours^12^, which should be sufficient for day to day use.

MMG is also more susceptible to movement artifacts than EMG, which can affect results substantially. This study limited these artifacts through static activities with no impact forces. Although devoid in this study, dynamic movements and impact forces were present in our previous work which were found to be manageable in software^12^.

Although EMG values seen here can typically be compared with other EMG results presented in literature which used wet electrode EMG, the MMG results can only be compared with other MMG articles which used a microphone for monitoring. Although alternative technologies can be used (accelerometers and hydrophones, etc.) it is important to note that a fair comparison can only been made between microphone studies due to differences in transducer parameters.

Furthermore, due to the small number of participants, as well as a disproportionate split of male to female, the results presented here are expected to be limited. Literature has shown that differences in gender show differing results in muscle properties and fatigue, which should be addressed in future work^35^.

The present protocol provided a useful model for using MMG to monitor exertion during different types of muscle activity (static, dynamic, eccentric, and concentric) but more strenuous contractile activity would have produced greater fatigue (e.g. higher force contractions or intermittent isometric contractions). An intermittent activity model was used to enable MMG responses to be studied under conditions of more severe and long-lasting fatigue than during sustained isometric contraction^32^. The purpose of the present study was to demonstrate the potential for the MMG/IMU device to be used in field settings for examining functional muscle activity. Here we show that in both dynamic and static conditions RMS and MPF from MMG are comparable to EMG measures.

The technology presented here could be beneficial for monitoring fatigue in clinics, which do not have access to equipment such as dynamometers or EMG, or in uncontrolled environments such as in the home. The amplifiers and size of technology associated with EMG in the present study would make it infeasible for use in pervasive settings. Conversely, the lightweight and portable MMG and IMU sensors were packaged into a single strap, with no external wires or preparation requirements.

The present findings demonstrate the ability to monitor muscle activity, as well as exertion, using low-cost and easy to use sensors, which do not require trained individuals or well-equipped laboratories.

The pervasiveness of the technology demonstrated opens the possibility of use in a far greater range of locations than current technology allows. Furthermore, the ease of application and use allows for a greater range of personnel to use such technology, which is currently reserved for professionals and clinicians.

The present results demonstrate how motion information can be used to segment and analyse specific periods of muscle data. However, these data were processed individually with no fusion of the datasets. Inertial, EMG, and MMG have the potential to be combined further, to provide greater insight into electro-mechanical relationships during muscular activity.

Other applications include the investigation for different types of fatigue (central or peripheral), due to the ease of applying these sensors to many places on the body, such as upper and lower limb.

With less environment restrictions this technology could be used in a variety of settings, including tracking muscle development in gyms or sporting environments, rehabilitation monitoring due to the unobtrusiveness of the sensors, which can be worn under clothing, and (despite not its target use) can still be used in clinical environments for assessment, including examining electro-mechanical uncoupling to study muscle physiology (including fatigue) during functional tasks. More extreme environments might also benefit from the technology, such as microgravity for monitoring astronauts, and under water during swimming, but would require specific technical considerations.

In conclusion, an IMU system was used for segmentation of the data into contraction phases, which could be used for identifying activities and training sets or analysis of contractions at specific periods. Furthermore, the present study compared an MMG sensor system against EMG for future use in pervasively monitoring muscle activity. The changes seen in EMG and MMG were compared against previous literature which confirmed the expected behaviour of the signals recorded in the present study. Squatting repetitions were identified and segmented into the three stages of contraction activity (entering, held, and exiting the squat) of the rectus femoris muscle using an IMU. The MMG/IMU package is inexpensive, easy to use, and completely wireless, allowing for use outside of a laboratory or controlled environment, during everyday activities.

## Methods

Five healthy adult participants, one female and four male, were recruited for this study. This research was approved by the Imperial College Research Ethics Committee (ICREC) and informed consent was obtained from each participant prior to completing the study. All methods and experiments were performed in accordance with relevant guidelines and regulations. All participants in this study gave explicit verbal and written informed content to be included in any identifiable or non-identifiable figures, videos, or other media for any form of publication purpose (including online open-access articles). Participants were asked not to perform any strenuous activities at least two days before the experiment to avoid the potential for any long-term muscle fatigue. We analysed contraction data from the rectus femoris while the participants performed a wall squat to exhaustion. Both MMG and EMG were collected concurrently with motion information from an IMU. The rectus femoris was chosen as the muscle of interest due to its superficial location and ease of achieving reliable sensor positioning, as well as being the subject of many other muscle-based squatting studies^36–38^.

**Figure 4:**
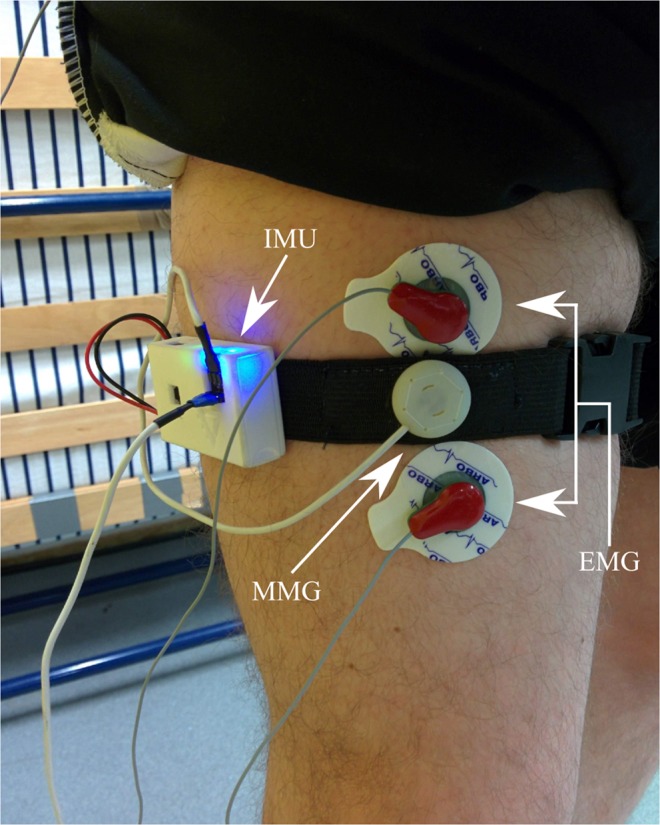
Sensor configuration for this study with MMG and EMG electrodes positioned over the rectus femoris muscle (quadriceps) 50% between the anterior spina iliaca superior and the superior part of the patella, and the IMU parallel to the line on the lateral side of the leg. The MMG sensor was sewn into the elastic strap and the Mylar membrane was resting upon the skin above the rectus femoris.

Prior to the study, the recording site was located over the right rectus femoris muscle on the front of the right thigh by measuring 50% of the distance between the anterior superior iliac spine and the superior pole of the patella, which is a known motor point^39^. The EMG electrode sites were shaved and abraded before adhesive attachment. The MMG device was placed at the 50% point, with EMG electrodes longitudinally on either side of the MMG, with 5 cm spacing between them (2.5 cm between EMG and MMG), similar to that seen in previous literature which monitored EMG and MMG concurrently^40,41^. The EMG reference electrode was placed on the anterior spina iliaca superior (right side). The IMU was placed on the lateral side of the right thigh. The MMG and IMU devices were secured to the thigh with an elasticated band and the Mylar membrane of the MMG sensor was resting upon the skin above the rectus femoris (Fig. 4).

The EMG system used was a g.tec g.BSamp biological amplifier with a g.tec g.GAMMAbox electrode driver (gain 1000x). The MMG sensor was custom made and consisted of a microphone (Knowles SPU1410LR5H-QB) contained within a sealed chamber with a Mylar membrane at one end which was placed against the skin atop of the muscle of interest. Any disturbances against the membrane (e.g. because of mechanical muscle movement) resulted in a pressure change within the chamber which the microphone detected and recorded. MMG was not amplified. The IMU device was custom made with a 2000° per second triaxis gyroscope (STMicroelectronics L3G4200D), a ±16 g triaxis accelerometer (Analog Devices ADXL345), a ±8 G triaxis magnetometer (Honeywell HMC5883L), and a −500 to +9000 m (sea level) barometer (Bosch BMP085). The IMU also provided a four channel analog to digital converter, which was used to collect both the EMG and MMG data. Both the MMG and IMU devices are described in greater detail in our previous work^12^.

MMG and EMG signals were sampled at 1 kHz using the IMU’s auxiliary port. The inertial components (gyroscope, accelerometer, and magnetometer) of the IMU were sampled at 50 Hz, however, only the accelerometer was used in this study. All data were transmitted wirelessly to a laptop via Bluetooth (class 1, V2.1). Data packets were transmitted over the universal asynchronous receiver/transmitter (UART) protocol at a baud rate of 230400 and contained a header byte and two checksum bytes at the start and end of a packet, respectively. The header byte indicated the type of data transmitted (IMU or auxiliary), while the checksum was used to determine successful transmission of the data. Data transmission was tested thoroughly during development of the IMU to minimise any latency or delay issues. No communication or dropped data packets were reported during this study.

In offline processing the MMG data were band-pass filtered between 2–100 Hz, whereas EMG was band-pass filtered between 10–500 Hz, using a 5^th^ order Butterworth filter. IMU data were smoothed using a moving average. All data processing was performed in MATLAB.

The study was divided into three stages. Participants performed unloaded squats to determine the muscle response before an exerting activity (stage one), exerted their muscles during sustained isometric activity with a wall squat (stage two), and then performed unloaded squats once more to determine muscle performance post-exertion (stage three). Participants were instructed in the correct squatting method prior to testing, with knee and hip flexion angles estimated. The stages are described in detailed below.

**Figure 5:**
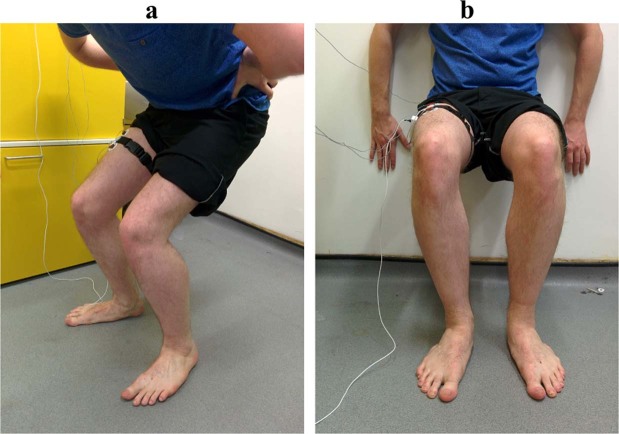
(**a**) Stage one and three — Five two-legged squats held for five seconds, with a five second pause between. Hands on hips, feet shoulder width apart. Squat angle performed to a knee flexion of 75°. **(b)** Stage two — Two-legged wall squat held for as long as possible. Hands flat against the wall with knee flexion at 90°. Feet shoulder width apart.
Stage Two: Following stage one, participants were immediately asked to perform the second task of a two-legged wall squat. This consisted of them squatting with their back against a wall, with their knees at a 90° flexion, hip flexion at 90°. Again, actual knee flexion was estimated using inertial information from the IMU. Feet were again in line with the shoulders, with the hands placed flat against the wall. The participant was asked to remain in this position for as long as possible. Once they could no longer hold the position, they were asked to stand up from the wall and sit on a chair provided (Fig. 5b).
Stage Three: To recognise any remaining fatigue in the muscle, each participant was given a ten-minute recovery period at this point before stage three began. Stage three was the same as stage one, with five two-legged squats performed to the same knee and hip flexion angles as before (Fig. 5a).

Stage One: Each participant was asked to perform five two-legged unloaded squats, where the squat was held for five seconds, with a five second pause between each. Feet were spaced shoulder-width apart. Hands were placed on the hips and participants were asked to squat to a 75° knee flexion and 90° hip flexion. Estimation of knee flexion was achieved by zeroing off the accelerometer data when standing to obtain a resting gravitational reading perpendicular with the horizontal plane. Hip and ankle flexion were not measured and only estimated visually (Fig. 5a).

### Changes Post-Exertion

**Figure 6:**
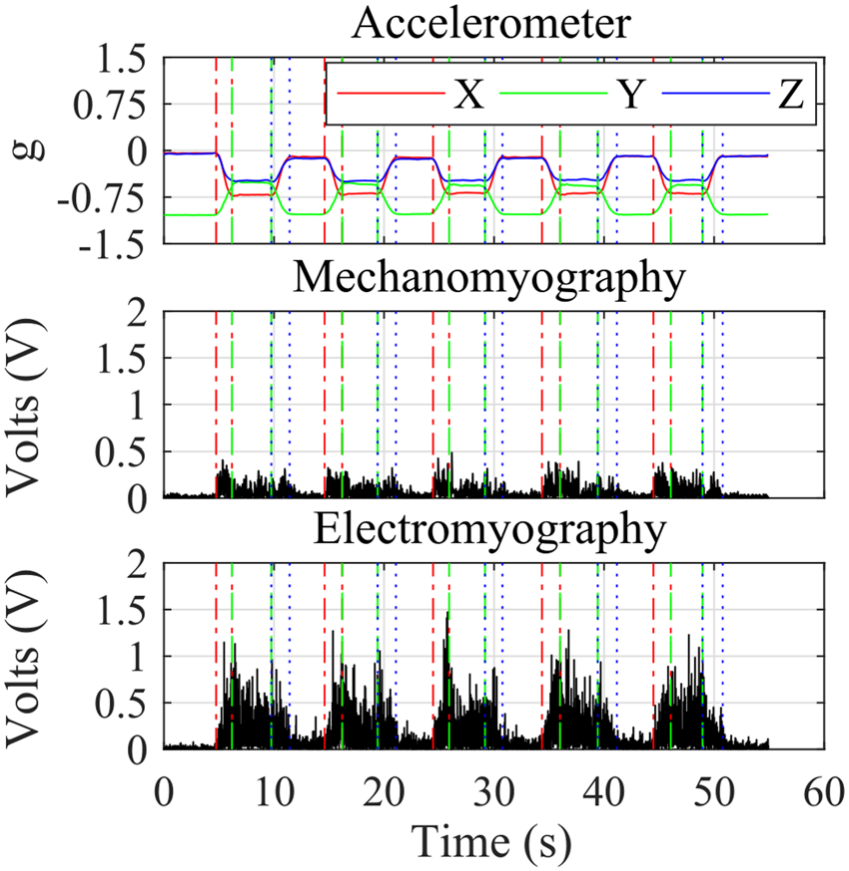
Example of muscle phase segmentation using accelerometer data from stages one and three of the exertion trial (figure showing data from participant 5, pre-exertion/stage one). Each repetition was detected and the start and stop points were segmented for entering (red dot-dashed line), held (green dashed line), and exiting (blue dotted line) the squat.

Stages one (pre-exertion) and three (post-exertion) were processed by first identifying data windows containing each of the five squat repetitions. The quadriceps contraction periods from each squat are defined as: entering (dynamic, eccentric, i.e. movement with muscle lengthening), held (static or isometric, eccentric) and exiting (dynamic, concentric, i.e. muscle shortening). The three stages of the squat were isolated and analysed using the accelerometer data. Data windows containing squats were extracted at points where the X plane accelerometer data exceeded a 10% increase and decrease in gradient from the stationary signal. Windows with a negative accelerometer gradient were taken as the period of entering the squat, whereas positive accelerometer gradients were taken as periods of exiting the squat. The periods between the entering and exiting windows were determined as the held (sustained) contraction portion of the squat (Fig. 6).

For both EMG and MMG, mean amplitude RMS and MPF were determined, which are often used in defining muscular activity. Amplitude (RMS) and frequency (MPF) data for both MMG and EMG were averaged across each of the five squats for each of the contraction windows (entering, held, exiting) per participant, for both pre- and post-exertion data sets. These data were then averaged across participants (n = 5) to obtain an average group result for the pre- and post- tests. RMS and MPF were represented as a percentage change between pre and post exertion, and a Wilcoxon signed rank test was used to determine significance of change.

### Changes During Exertion

**Figure 7:**
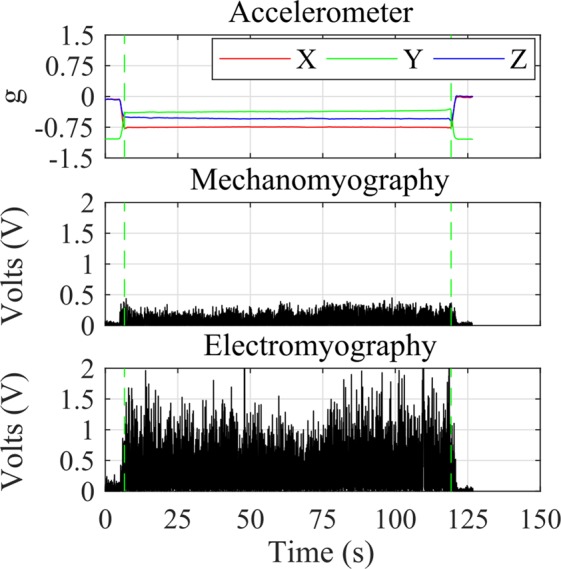
Example of muscle phase segmentation using accelerometer data from stage two of the exertion trial (figure showing data from participant 5, during exertion/stage two). Start and stop points were segmented for the held portion of the wall squat (green dashed line).

Stage two (during exertion) isolated the single wall squat in the same way as stages one and three; by detecting changes in accelerometer gradient. However, this stage only processed and analysed the period during the held contraction (Fig. 7).

The held period was divided into one second, non-overlapping windows, and each window had its RMS and MPF calculated, for both EMG and MMG data. Participant data were then grouped and the mean RMS and MPF was calculated.

Linear regression analysis was performed on the RMS and MPF change over time for both MMG and EMG, and Pearson’s correlation coefficients (*r*) and statistical significance (*P*) were calculated. Furthermore, linear slope gradients (*b*) and percentage change from the first (fresh muscle) and last (tired muscle) 10% of the held contraction window were also determined.

## Data Availability

The datasets generated and analysed during the current study are available in a Zenodo repository, 10.5281/zenodo.1299882.
